# Implementation of an integrated preoperative care pathway and regional electronic clinical portal for preoperative assessment

**DOI:** 10.1186/1472-6947-14-93

**Published:** 2014-11-19

**Authors:** Matt-Mouley Bouamrane, Frances S Mair

**Affiliations:** Institute of Applied Health Sciences, University of Aberdeen, Aberdeen, Scotland, UK; University of Glasgow, College of Medical, Veterinary and Life Sciences, Institute of Health & Well-Being, Glasgow, Scotland, UK

**Keywords:** *(Mesh)*, Medical informatics applications, Information systems, Integrated advanced information management systems

## Abstract

**Background:**

Effective surgical pre-assessment will depend upon the collection of relevant medical information, good data management and communication between the members of the preoperative multi-disciplinary team. NHS Greater Glasgow and Clyde has implemented an electronic preoperative integrated care pathway (eForm) allowing all hospitals to access a comprehensive patient medical history via a clinical portal on the health-board intranet.

**Methods:**

We conducted six face-to-face semi-structured interviews and participated in one focus group and two workshops with key stakeholders involved in the Planned Care Improvement (PCIP) and Electronic Patient Record programmes. We used qualitative methods and Normalisation Process Theory in order to identify the key factors which led to the successful deployment of the preoperative eForm in the health-board.

**Results:**

In January 2013, more than 90,000 patient preoperative assessments had been completed via the electronic portal. Two complementary strategic efforts were instrumental in the successful deployment of the preoperative eForm. At the local health-board level: the PCIP led to the rationalisation of surgical pre-assessment clinics and the standardisation of preoperative processes. At the national level: the eHealth programme selected portal technology as an iterative strategic technology solution towards a virtual electronic patient record. Our study has highlighted clear synergies between these two standardisation efforts.

**Conclusion:**

The adoption of the eForm into routine preoperative work practices can be attributed to: (i) a policy context – including performance targets – promoting the rationalisation of surgical pre-assessment pathways, (ii) financial and organisational resources to support service redesign and the use of information technology for operationalising the standardisation of preoperative processes, (iii) a sustained engagement with stakeholders throughout the iterative phases of the preoperative clinics redesign, guidelines standardisation and the eForm development, (iv) the use of a pragmatic and domain-agnostic technology solution and finally: (v) a consensual and contextualised implementation.

**Electronic supplementary material:**

The online version of this article (doi:10.1186/1472-6947-14-93) contains supplementary material, which is available to authorized users.

## Background

Admitting patients the night prior to surgery was until recently the traditional standard pathway for surgical hospital admission in NHSScotland. However, the routine identification of patients unfit for surgery on admission often led to late operating theatre cancellations, a waste of clinical resources and multiple hospital admissions. In response to the identification of these sub-optimal practices, combined with national requirements to increase the ratio of day-case surgery, alternative admission routes were developed and led to the development of dedicated surgical pre-assessment clinics [1]. However, the fragmentation of preoperative assessment (POA) services and a lack of standard processes for assessment and documentation introduced further issues, including: a lack of a coherent assessment framework across services, unjustified variations in clinical practices, a range of obstacles to seamless information sharing among the multi-disciplinary team (MDT) and a lack of service performance evaluation and auditing.

With an estimated patient population of 1,210,254 in 2011, the NHS Greater Glasgow and Clyde (GGC) health-board is by far the largest health-board in Scotland and one of the largest in the U.K. [2] Between 2007 and 2009, NHS GGC undertook to streamline, standardise and integrate preoperative assessment processes as part of the Planned Care Improvement Programme (PCIP) [3, 4]. The development of standard preoperative processes across the health-board was later instrumental in allowing the implementation of an electronic Integrated Care Pathway (ICP/eForm) to support documentation and information-sharing tasks across the MDT. All the NHS GGC hospitals can now access the preoperative eForm via a clinical portal on the health-board intranet.

This study describes the policy context leading to the development of standard POA processes in NHS GGC and the eHealth infrastructure within which the electronic POA ICP was integrated. We then used qualitative methods to analyse the perspectives of key stakeholders involved in the rationalisation of surgical pre-assessment clinics (PACs) in NHS GGC and the ICP design, development and implementation. Using Normalisation Process Theory – an explanatory framework that specifies important mechanisms and implementation processes within complex interventions in the health services [5] – our analysis focuses on identifying the complex sociotechnical factors that have influenced the successful adoption of the electronic preoperative ICP across NHS GGC in order to inform future implementations in this sphere.

## Methods

### Data collection

A RATS (Relevance - Appropriateness - Transparency - Soundness) check-list describing our qualitative research methods in detail is provided as an Additional file 1 (ANNEX I). Ethical approval for this study was obtained in February 2011 from the University of Glasgow College of Medicine ethics committee. The results presented in this study relate specifically to the data collected as part of the review of PACs in NHS GGC. As part of a study of information management processes in the patient surgical pathway across NHSScotland, we visited preoperative clinics in all 14 territorial health-boards of Scotland, and conducted semi-structured interviews with primary care practitioners between February 2011 and January 2013. In the course of the national study, we interviewed 25 general practitioners and carried out 45 interviews with members of the preoperative multidisciplinary team and 4 members of the eHealth programme and NHS IT team [1, 6–8].

- **Study sample:** Between May 2011 and February 2012, we conducted 6 face-to-face, in-depth, semistructured interviews and one focus group with stakeholders involved in the preoperative ICP development in NHS GGC. Contacts were made with NHS GGC via email in order to identify key individuals behind the development and implementation of the electronic preoperative clinical portal. Three main stakeholders were identified and contacted by email. We provided background information on the purpose of this study [7] and suggested arranging a date for an interview. All the three stakeholders approached agreed to take part in an interview. These were:

**- eForm 1:** a member of the NHS GGC electronic patient record programme (EPR) eForm team involved in the development of design requirements and technical specifications for the preoperative ICP,

**-Anaesthetist 1:** a consultant anaesthetist involved in the consensus process which led to development of the structured clinical content of the preoperative ICP, including the selection of guidelines underpinning the context-dependant, adaptive behaviour of the eForm.

**-POA nurse 1:** a senior nurse involved in the PCIP review of the NHS GGC PACs and the dissemination of information relating to the programme implementation across the health-board. In addition, the nurse was involved in the eForm user-testing, reporting user requirements and change requests to the eForm development team.

In addition, to these 3 interviews, we also conducted in February 2012 a case-study at one preoperative clinic in an NHS GGC Acute Care Hospital (ACH). On that occasion, we interviewed the service lead nurse and 3 nurses who worked in the clinic. The nurses were routine users of the preoperative eForm during patient assessment.

All the participants provided explicit, signed informed consent to participate in the study at the time of the interview and agreed to have the interviews audio-recorded and transcribed. Interviews duration ranged from over 20 minutes to over an hour and 20 minutes, with a mean duration of approximately 43.5 minutes per interview. The interviews were semi-structured and open-ended in order to allow the interviewer or interviewee to elaborate on unanticipated and potentially valuable information with additional questions, and probe for further explanation [9].

In addition to the above interviews, a focus group organised by the NHS GGC POA team and members of the EPR eForm programme took place in August 2011 in one of the NHS GGC ACHs. The aim of this meeting was to present the implementation of the electronic portal and POA eForm to a nursing, IT and clinical management delegation from NHS Tayside. A researcher (M-M.B) was invited to attend the meeting. The other participants in this meetings included 2 members of the NHS GGC EPR eForm project and – from NHS Tayside – a nursing manager, 2 POA nurses and a member of the ACH IT department. The meeting duration was just slightly under 3 hours and was digitally audio-recorded by the researcher with the explicit consent of all participants.

- Finally, one researcher (M-M.B.) was invited to attend 2 forums organised by the NHS GGC EPR programme. These workshops lasted for a full-day and aimed to provide a platform to share experiences on a range of eHealth implementations across NHSScotland. Participants were members of the eHealth programme and NHS staff from various health-boards. This was an opportunity for the researcher to take notes and discuss the stages of implementation of the clinical portal in NHS GGC and other health-boards with a range of active stakeholders.

### Data analysis

Over 7 hours and 15 minutes of audio recording were transcribed verbatim and qualitatively analysed [9]. We used process-mapping techniques to model POA processes in NHS GGC [10–12]. We then used Normalisation Process Theory (NPT) as a conceptual framework to interpret the factors which were identified as facilitating or hindering the work of the members of the MDT. NPT is concerned with the social organisation of the work (implementation) of making practices routine elements of everyday life (embedding) and of sustaining embedded practices in their social contexts (integration) and was developed particularly in response to the evidence, which suggested that electronic health implementation, embedding and integration are difficult to achieve in practice [13–15].

The interview transcripts were analysed and coded by one researcher (M-MB). The two co-investigators (MML & FSM) then discussed the coding framework used on the transcripts in “coding clinics" to ensure a consistent approach to coding and the validity and robustness of the proposed coding framework.

This thematic framework was designed on the four key generative mechanisms of NPT: coherence; cognitive participation; collective action and reflexive monitoring [13, 14].

- **Coherence:** refers to the work of making a complex intervention hold together and cohere to its context, how people “make sense" or not of the new ways of working.
- **Cognitive participation:** is the work of engaging and legitimising a complex intervention, exploring whether participants buy into and/or sustain the intervention.
- **Collective action:** examines how innovations help or hinder professionals in performing various aspects of their work, issues of resource allocation, infrastructure and policy, how workload and training needs are affected and how the new practices affect confidence in the safety or security of new ways of working.
- **Reflexive monitoring:** is the work of understanding and evaluating a complex intervention in practice, and how individuals or groups come to decide whether the new ways of working are worth sustaining.

## Results

We present our results along the 2 main themes which have emerged from the qualitative data analysis: the development of standard preoperative processes in the NHS GGC health-board and the subsequent implementation of the electronic integrated care pathway for surgical pre-assessment. We then illustrate our findings in context through a case-study conducted at the PAC of one of the NHS GGC ACHs. For purposes of readability, we have included essential excerpts of interviews to support our data analysis within the main article and refer to relevant sections of the Additional file 2 for additional quotes where appropriate.

## Standardisation of pre-operative processes across NHS greater Glasgow and Clyde

### Aims of pre-operative assessment

POA for elective surgery is essentially a clinical triage process, aiming to identify potential risks of perioperative complications. There is overall a lack of evidence-base underpinning the key steps needed for the provision of effective pre-assessment services [16–19]. An important element of our study therefore was to elucidate health professionals’ *beliefs* around the aims of pre-operative assessment (see Additional file 2: Appendix-1 for interviewees’ quotes on their opinions regarding the aims of POA). In particular, how members of the MDT felt that an optimum PAC should be organised and operationalised in practice. Key tasks identified include: *collecting a complete patient medical history and performing relevant screening tests for appropriate patient risk assessment, coordinating the patient pre-admission plan, including collating the results of screening investigations and sharing information accordingly with relevant members of the MDT, coordinating further referrals as necessary, informing the patient of the risk associated with surgery and obtaining informed consent, optimising patient’s fitness for surgery and making optimum use of clinical resources*.

During POA, the nurses not only play an important role in collating information from patients and various other sources, but also in coordinating screening investigations, anaesthetic reviews for complex patients, the planned admission routes and liaising with other services for the patient pre-admission management. It is therefore essential that they have efficient means to coordinate these tasks as well as being able to share relevant information with a wide range of other health professionals, such as other members of the MDT, other departments within the hospital or even other services within the NHS, such as the primary/community care teams [1].

- **POA Nurse 3**:*“ We basically take their basic information, their past medical history, anything that’s current and pertinent to their admission, really. And we collate all that and if we need to let the anaesthetist... the consultant know, we do that [...] so it sorts of encompass all specialities at one time or another. And so... and then, like I say: take all that information and decide whether the patients are of a decent risk for surgery and that we wouldn’t need a referral to anybody else, we just put them through as suitable. If they’re questionable, we would contact the appropriate person – be that the anaesthetist or the surgeon or both – and find out from them whether they believe the patient’s suitable and for what admission they’re suitable."*

### The planned care improvement programme review of pre-assessment clinics

In 2006, the Planned Care Improvement Programme aimed to improve the flow of patients along their healthcare pathways through sustainable clinical systems improvement [3]. PCIP required all health-boards to develop 3 year implementation plans, focusing on 5 key, high-impact change priorities: *(i) improve referral and diagnostic pathways, (ii) treat day surgery as the norm, (iii) actively manage admissions to hospital, (iv) actively manage discharge and length of stay and (v) actively manage follow up*. The third PCIP priority (i.e. active admission management) was a key driver for the development and streamlining of PACs across NHSScotland. NHS Borders, Dumfries & Galloway, Orkney, Shetland and Tayside developed new PACs while NHS GGC, Highland, Lanarkshire, Lothian, Tayside and Western Isles undertook to streamline and standardise pre-assessment procedures and services [4].

- **Nurse 1**: (about the development of integrated PACs in NHS GGC): *“It came out as part of the Planned Care Improvement Program and Managed Project for Admissions... and one of the things that we were looking for was to increase day-surgery rates and same-day admission beds... and basically one of the things that they thought could facilitate that was: if everybody has been pre-oped ’cause if everybody had been pre-oped, then they could come in the morning of their surgery."*

Thirty distinct pre-assessment services, in a variety of hospitals and clinical departments, were identified across NHS GGC during the course of the PCIP review into preoperative practices within the health-board. Not all pre-assessment services were based indedicated clinics and some were providing other clinical services in addition to surgical pre-assessment. Services were fragmented, with no standardised preoperative assessment guidelines and documentation across the various services, with each using locally developed and specialty-specific processes [4]. In addition, a substantial number of patients were still not being systematically assessed prior to surgery.

- **Nurse 1**:*“...so then it was looking at: ‘well how many different pre-ops are there to do the work in?’... and they’ll be using different documentation and they all using different guidelines and this is how this came about. So we think: ‘Greater Glasgow and Clyde: it’s a generic pre-op on top of Greater Glasgow and Clyde’. Not just south and the north (of the city): Clyde as well. They use the same documentation. It means they’re using the same guidelines."*

**Figure 1:**
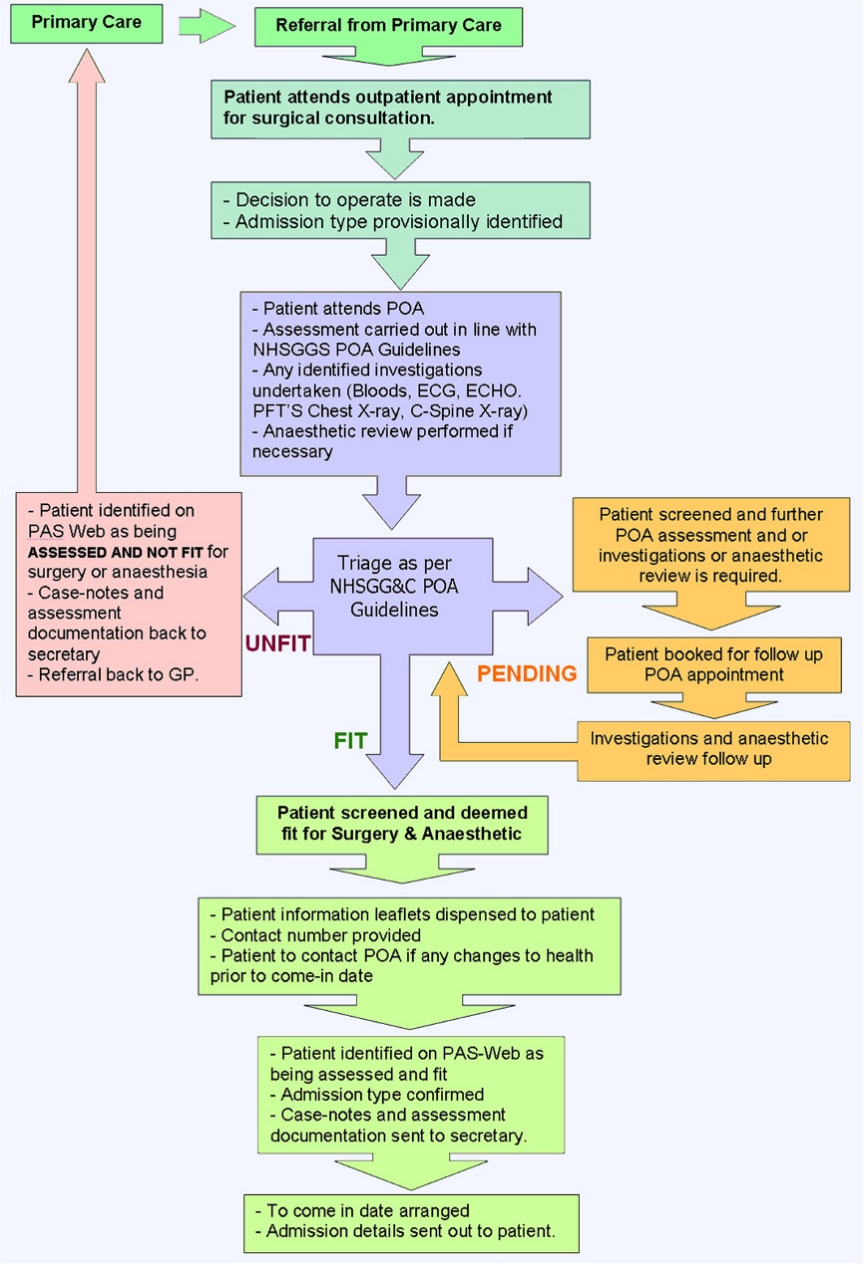
**NHS greater Glasgow and Clyde, standard preoperative patient pathway.**

The PCIP/PAC review undertook a broad consultation with stakeholders, senior management, clinical leads and front-line staff. Anticipated organisational benefits of the PAC review included: *the development of standardised POA patient pathways and processes across the health-board (see Figure*1*), improved hospital admission and discharge processes, the implementation of evidenced-based guidelines, a reduction of inpatient admissions and increase in day-case surgery rates, a reduction of non-attendance and surgical cancelations, improved utilisation of hospital resources, improved operating theatre utilisation, a reduction in hospital stay and cost savings*.

The consultation led to the development of: (i) a PAC staff directory across the health-board, (ii) a training needs analysis documents, (iii) a standardised POA data set, (iv) standard POA clinical guidelines and (v) a communication strategy for explaining the rationale behind the POA guidelines standardisation across the health-board.

**Pre-operative guidelines consensus building:**

The PCIP explicitly excluded allocating resources from the programme to ICT implementations, with an emphasis instead on services redesign and whole system changes. Innovative electronic health systems implementation within NHSScotland is the remit of the Scottish Government Health & Social Care Department eHealth programme [20]. However, the standardisation work undertaken as part of the PCIP was instrumental in laying the ground for the future deployment of the preoperative eForm: by providing an agreed, structured POA documentation, as well as a framework for data collection and decision support functionalities in the form of consensual protocols and guidelines (see Additional file 2: Appendix-2).

## Electronic preoperative integrated care pathway implementation

### The eHealth clinical portal programme

Multiple patient medical documents are created at each stage throughout the patient journey through the health services: from an initial primary care encounter, through referrals to specialist services, transfer of care and discharge back to community care [1, 6–8]. This often leads to multiple inefficiencies in information management, including: multiple instances of data elucidation, data duplication, missing information as well as multiplying the risks of erroneous data entry and transfer, contributing to patients’ and clinicians’ frustration, increased cost and potential adverse events and patient harm. Efficiency and quality improvement in the storage and transfer of patient data was identified as a strategic priority by the eHealth programme, due to structural inefficiencies and the high costs associated with existing data storage and transfer systems.

- **eForm 1**:*“The cost of storage, transport, filing, medical records is in excess of 2.5 million (£) for us alone (NHS GGC). Take that across all the health-boards and it’s a lot of money. We adopted a bottom-up approach because we wanted to work from... [...] If you want a job done, you’ve got to engage everyone that’s required but you’ve also got to start with: where’s the bulk of the information coming from? How’s it getting there?"*

Even when held in electronic format, patient medical information typically remained stored in service-specific electronic repositories – i.e. data ‘silos’ – and the transfer of patient data across services was often inefficient and suboptimal. The eHealth programme aimed to improve the timely access to electronic patient records through an efficient and coherent electronic infrastructure across health-boards. The strategic vision was: (i) to move away from specialist-specific ‘niche systems’, (ii) to promote rapid and incremental implementations building on previous successful deployments rather than attempting potentially risky and costly whole IT system changes and (iii) selecting portal server technology as an iterative strategic technology solution towards a virtual electronic patient record [21–24].

Portal technology is an internet-based content aggregation solution whereby a web interface provides information and functionalities as a single point of access by integrating heterogeneous systems and reusing existing functionalities instead of commissioning new, multiple, duplicate information systems [25]. The electronic portal is deployed upon a standards-based, layered architecture, and is combined with a scanning and electronic document management (EDM) system. Due to the variations in IT capacity and infrastructure across health-boards, the implementation of clinical portals is phased and incremental. NHS GGC and NHS Tayside were the first health-boards to deploy the technology and the eHealth Clinical Portal programme aims to ensure that other health-boards incrementally reach a baseline of clinical portal functionalities. The funding for the development of the clinical portals comes from the Scottish government eHealth programme and not from local hospitals. The total cost of portal technology development across NHSScotland was estimated in 2010 to be between £10 to £15 million^a^.

- **Integration within the national eHealth implementations:**

**Figure 2:**
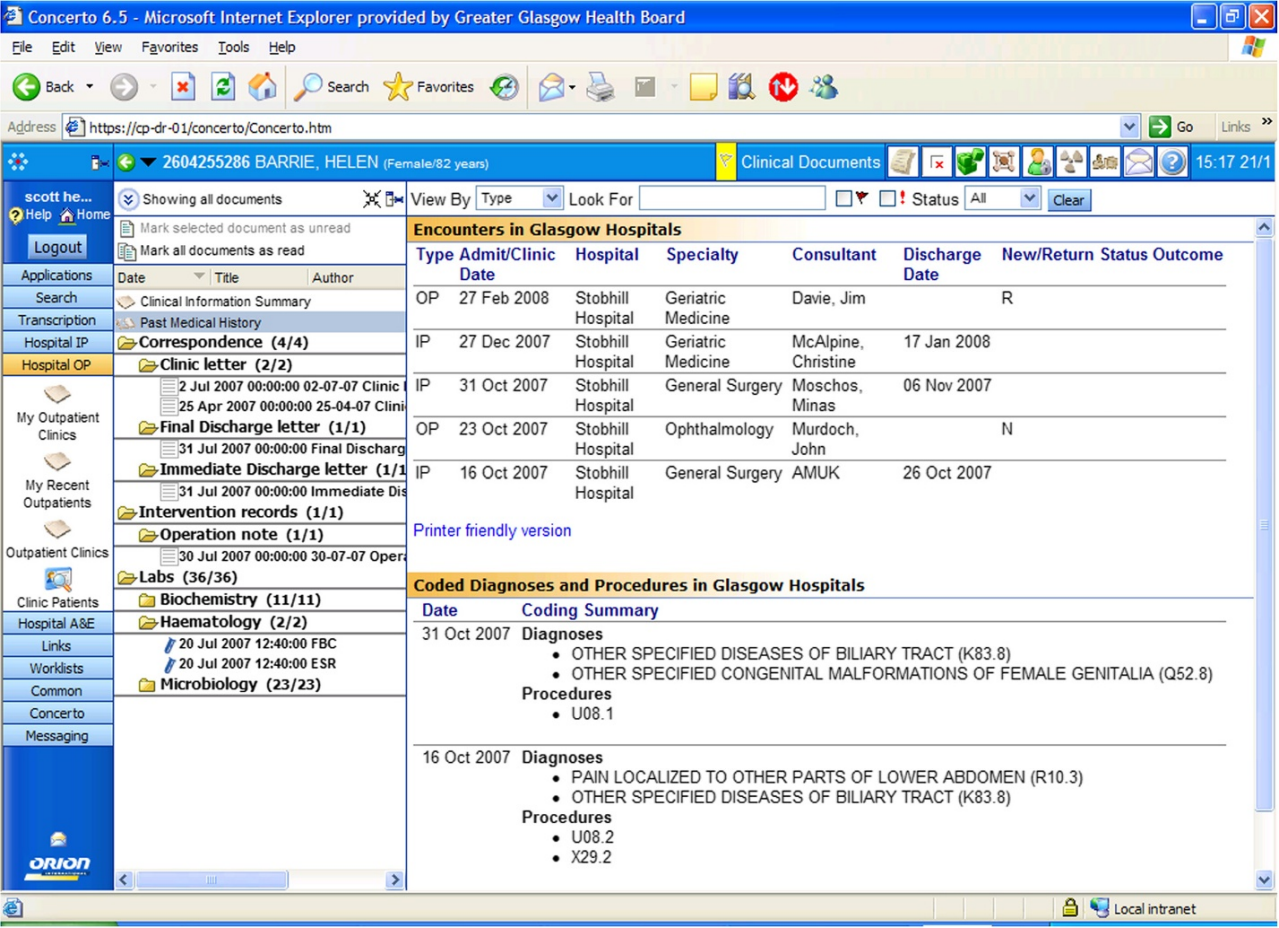
**NHS greater Glasgow and Clyde electronic clinical portal.**

By aggregating clinical data held in heterogeneous systems and databases (hospital information systems, national and health-board electronic repositories), the clinical portal allow clinicians to access a wide range of patient medical information, including: medical history, test results, clinical letters, medication list and other relevant patient information from a single electronic window (see Figure 2 and Additional file 2: Appendix-3). Role-based access control grants individuals various levels of information access depending on clinical roles, in order to comply with statutory data protection legislation and the NHS information governance framework.

**Figure 3:**
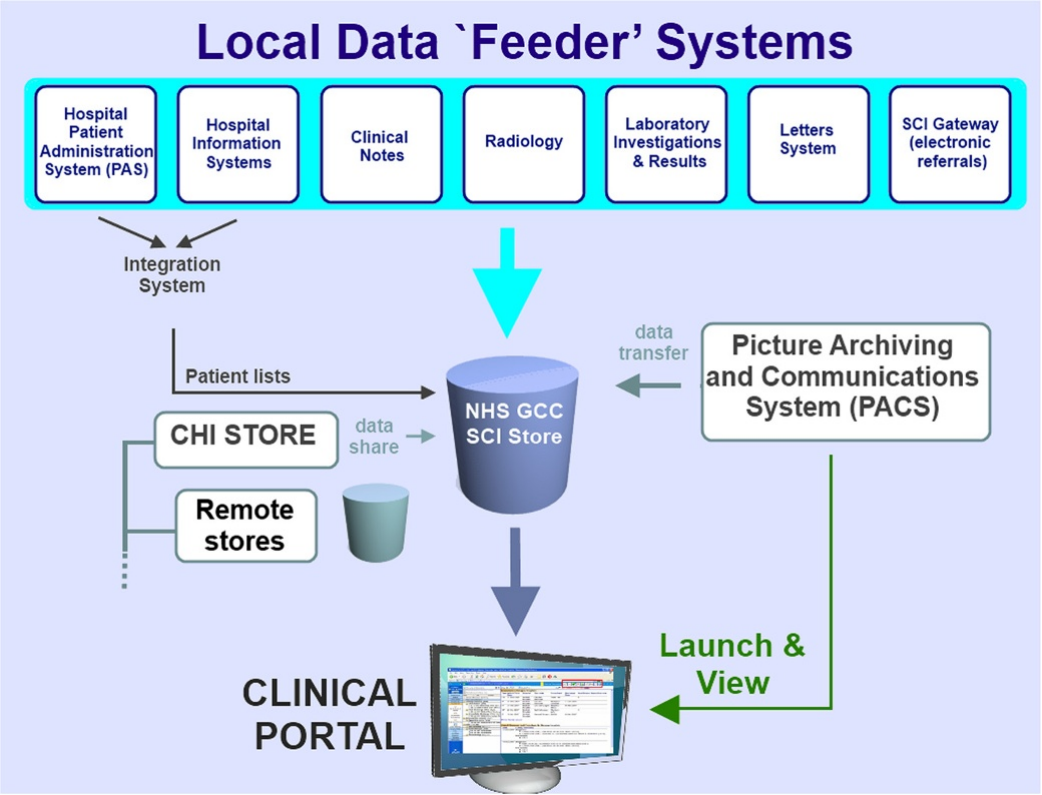
**NHS GGC electronic clinical portal & eHealth systems architecture.**

The portal can provide access to both national and regional electronic data repositories. Nation systems accessible include the Community Health Index (CHI) Store, the Picture Archiving and Communications System (PACS), the electronic pharmacy (ePharmacy) system implementations and the Emergency Care Summary [23] (see Figure 3 and Additional file 2: Appendix-4).

- **Community Health Index**: The CHI number provides a single, central, national patient identifier across NHSScotland. CHI identification is instrumental in enabling the aggregation of patient data from multiple heterogeneous clinical data repositories and systems.

- **eForm 1**:*“[...] So if you launch the portal, you can search for your patients individually using a CHI, their name and date of birth or their unit number for that hospital because SCI Store holds that patient’s CHI information and also holds all the case-note numbers against that patient. So regardless of where you’ve been... you can see where you get a case-record number. And portal’s looking and given the view of: ‘well here’s everything that I know about that patient’. [...] Portal has the ability to store your user ID and password, so you’re storing that and it’s passing through those credentials to the other systems. So it’s cutting down that ‘in and out’ of systems. It’s trying to make it slick and functional, so that users are getting the benefit of seeing everything in the one place instead of having to access it all separately and individually."*

- **Picture Archiving and Communications System**: The PACS provides access to the national archive of electronic images and radiological reports, shared across NHSScotland. The PACS allows radiology images, such as X-rays and scans, to be stored electronically and viewed remotely on digital screens.
- **ePharmacy**: the national programme providing the IT infrastructure allowing the electronic transfer of prescriptions (ETP) in NHSScotland [26].
- **Emergency Care Summary**: The ECS provides summary information for unscheduled (i.e. emergency) care, including CHI number, basic demographic information, registered GP as well as allergies and prescribed medication. This information is updated on a daily basis, pulled directly from primary electronic medical records systems [6].

In addition to the above, the electronic portal can also access information from the regional secondary central electronic data repositories, SCI Store. SCI Store is implemented in every health-board and provide clinicians with secure access to patient information, including patient demographics, laboratory results and investigation reports, treatment logs and admissions, referral, handover, transfer and discharge documents.

**NHSScotland clinical document indexing standard:**

Needless to say that the implementation of clinical portal technology and electronic document management requires a coherent and consistent indexing standard across NHSScotland so that clinical documents can be stored, accessed and retrieved effectively at the point of care to support information sharing and to reduce the risk of documents being lost or misplaced. A new national standard was released in 2012 setting an agreed list of clinical document indexes, including document types, sub-types and the metadata recommended to be associated with a clinical document for storage, sorting and retrieval [27].

### Development of an electronic preoperative integrated care pathway

The implementation of a preoperative Integrated Care Pathway constituted the next major step towards the integration of surgical pre-assessment processes across the health-board. The ICP development was initiated in 2007 by the electronic patient record programme and the eForm was rolled out in 2009 on the NHS GGC intranet (electronic portal).

The iterative stages of design and development included:

- (i) clinical requirement gathering with stakeholders,
- (ii) POA ICP design specification & system development
- (iii) iterative user-testing
- (iv) final implementation and roll-out.

**• (i) Clinical requirement gathering with stakeholders:**

Anaethetist 1 developed in consultation with colleagues (also anaethetists) across the health-board a structured POA questionnaire. It is important to highlight that the eForm was from the outset designed as a dynamic questionnaire and not a static one. The eForm adapts on-the-fly to previous clinical data entry, using underlying multi-level conditional branching, prompting context-specific clinical decision support functionalities for the nursing staff. This adaptive behaviour has previously been described elsewhere as a desirable feature of surgical pre-assessment information systems due to the complexity of information elucidation tasks in POA and the importance of conducting patient-centric screening, risk stratification and care management [28–35]. Consensus guidelines agreed across the health-board through the PCIP programme, and including guidelines developed by the Scottish Intercollegiate Guidelines Network (SIGN) [36] are embedded in the ICP. Importantly – as a result – the eForm not only enabled the standardisation of POA data collection tasks but also *promoted the standardisation of nursing clinical processes across the participating sites*.

- **Anaesthetist 1**:*“[...] we’ve got guidelines for tests which are based upon, either co-morbid diseases, for example, if you know... through the guidelines, they triggered certain questions... that will then be an automatic screening investigations, which – I mean the nurse can decide herself – but the form will complete it. So as they trigger, if they say ‘yes’ to... they say ‘yes’ to something like: ‘I get chest pain when I walk up a hill’, then automatically the interface summary form that I showed you, there will be a tick-box, with tick ‘yes’ for an ECG. These have all been decided at a time when we were developing up the guidelines and so that... that’s all standardised throughout Greater Glasgow and Clyde."*

In many cases, nurse assessors will still need to rely upon their professional experience and clinical judgement in order to decide how individual patients need to be managed depending on the planned surgery and their personal circumstances (see Additional file 2: Appendix-5). Having direct access to relevant past-medical history information and the ability to share information among the members of the MDT can therefore help improve individual patient case-management. Yet, the provision of comprehensive decision support functionalities remains currently limited by the complexity of the medical domain and the lack of robust evidence in the field [35].

**Figure 4:**
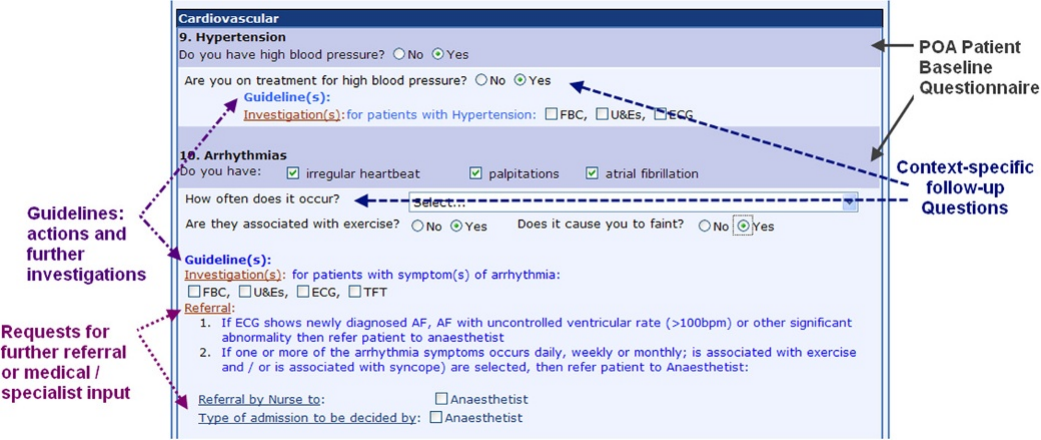
**Sample of NHS GGC Nursing Preoperative eForm questionnaire, with embedded decision support functionalities, including: (i) questionnaire conditional branching, (ii) clinical guidelines and advice for further investigations and (iii) requests for further referrals or medical input].**

- **(ii) POA ICP design specification & system development:**The POA ICP was designed so it could be easily completed by nurses with minimal need for typing. The eForm is therefore mostly composed of check-boxes. As we have highlighted previously, morbidity-specific decision support functionalities are embedded within the system, so that if the patient is found to have certain conditions potentially associated with risks of perioperative complications, then additional prompts are automatically triggered. These functionalities are illustrated in the sample POA eForm seen in Figure 4.

- **Anaesthetist 1**:*“[...] we came up with the structured questionnaire, and then its multiple level of questions and we then came up with the guidelines so that they were answered a particular way, the nurses would know how to do it. A lot of it is straight-forward, a lot of simple data collection, but there’s quite a few questions in there which are key questions, which if the patient answers positively to them, then a trigger a... follow-up questions, and then that will then lead to either an automatic referral by the nurses... for example: if they had a heart attack 6 weeks ago, the nurses wouldn’t bother contacting me: they would send him off to the cardiologist."*

**• (iii) Iterative user-testing:**

The ICP was iteratively tested by the target end-users (POA nurses, see Additional file 2: Appendix-6). A key design feature which emerged from the user-testing phase was the design of *two distinct* forms for assessment:

(i) ‘First-line’ document: a stream-lined form for the routine and rapid assessment of healthy and fit patients
(ii) a comprehensive assessment for complex patients, either with significant morbidities or scheduled for major (i.e. higher) risk surgery.

These two distinct forms are essential to improve the efficiency of POA as previous studies have clearly indicated that specific types of patients account for most post-operative adverse events. A study by Pearse et al. in 2006 concluded that while 12.5% of surgical operations are performed on high risk surgical patients, this population accounts for more than 80% of post-operative deaths [37]. A report by the National Confidential Enquiry into Patient Outcome and Death (NCEPOD) in the U.K. also highlighted that patients undergoing surgery are increasingly older, often with complex chronic morbidities requiring optimum preoperative planning [38]. The report clearly highlights the importance of high quality POA to ensure the early identification and effective clinical management of “higher risks" patients, in order to reduce surgical mortality rates. Thus, if time and resources can be saved during the evaluation of routine patients, additional resources may be reallocated to those requiring more extensive management. In addition, we have also reported elsewhere that static and uniform data collection instruments for all patients, regardless of their personal circumstances, were often perceived by the MDT as being time-consuming and cumbersome [1].

- **Nurse 1**:*“[...] there’s two, there’s a first-line pre-op which is a quick one for day-cases and people that don’t have much co-morbidities and then there’s the full pre-op assessment form [...] ‘First-line’ document was one of the best things that came out because you were spending a lot of time with young, fit people who were just going for day-surgery... asking them a whole load of questions and they’ll answer ‘no’ to them all. So you’ve got a quicker document, and it was a much easier[...] You do get people answering ‘yes’ to every single question, so you could imagine it would be a bit time consuming. [...] Young fit people who don’t have any co-morbidities, that’s a day-case, only for day-case. 23-hour don’t use this or inpatients at all. And it’s got... The anaesthetists have decided on the most pertinent questions that they would want to know regarding somebody that’s going for an anaesthetic so you’re not missing out diabetes, you’re not missing heart, stroke anything like that. The most important ones are there."*

**• (iv) Final implementation and roll-out:**

Once a stable system had been approved by Nurse 1, the eForm was then rolled out in 2009 in a NHS ACH, where it was initially used by 5 nurses in the hospital. Within a period of approximately 8 months, other PACs in NHS GGC also started using the eForm. The system remains iteratively updated by the EPR eForm team as the nurses provide new end-user specifications. As with any IT implementations, many issues will not come to light until the system is actually used as part of routine practice (see Additional file 2: Appendix-7).

**Figure 5:**
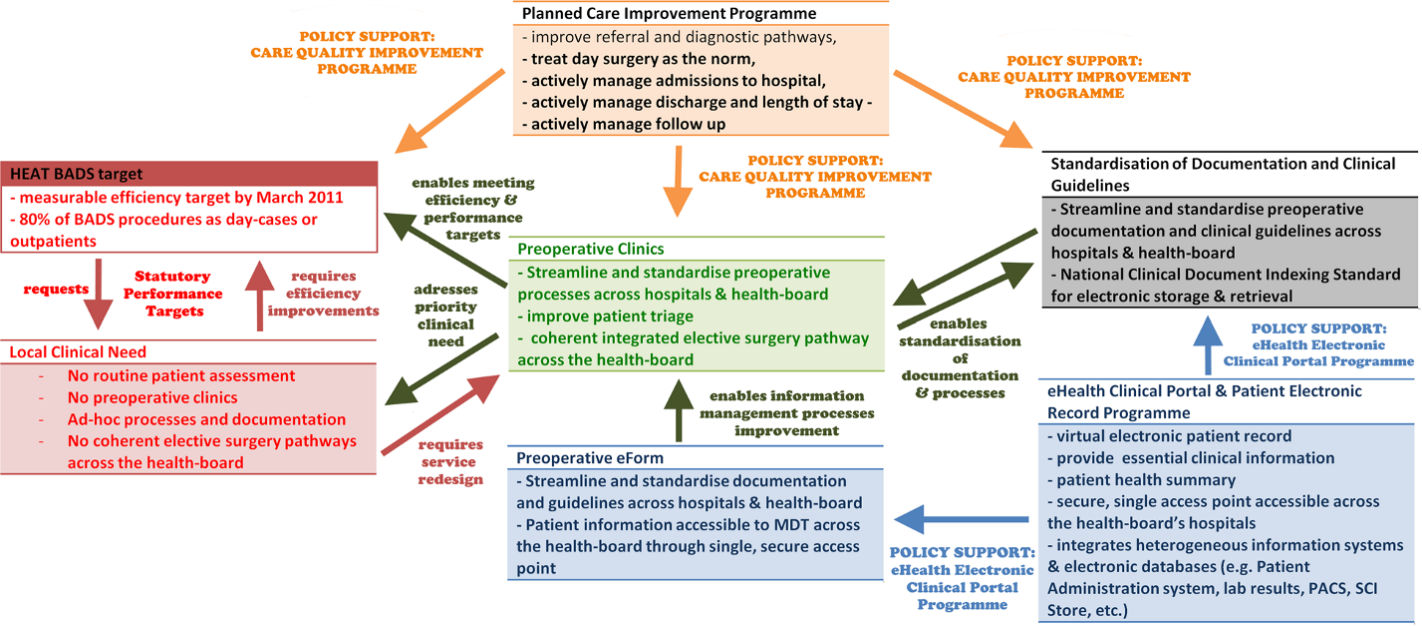
**Synergies between quality improvement & eHealth programmes: the Planned Care Improvement Programme (PCIP) and Electronic Patient Record (eForm) Clinical Portal programme.**

Approximately 20,000 patients were assessed through the POA eForm within 18 months of the initial implementation. In August 2011, the NHS GGC eForms project manager reported that the system was routinely used by 90 preoperative nurses and 25 anaesthetists across 9 sites of the greater Glasgow and Clyde health-board, including all the major acute care hospitals: Stobhill hospital, the new Victoria Infirmary, Glasgow Royal Infirmary, the Western Infirmary, Gartnavel hospital and the Southern General Hospital. Approximately 41,000 patient preoperative assessment had been completed through the electronic portal between March 2009 and July 2011. The estimated number of new assessment via the portal was approximately around 400 to 500 a week in 2011. The latest available figures (31-01-2013) reported that the POA ICP was being used by 145 nurses and 35 anaesthetists and that 90,404 assessments had been completed since March 2009, with an average of 750 to 800 new assessments per week in January 2013.

**• Efficiency outcomes indicator - BADS procedures**

The British Association of Day Surgery (BADS) has developed a Directory of Procedures which now includes over 200 recommended Day and Short stay surgical procedures, coded and categorised by surgical specialty [39]. A report by Audit Scotland in 2004 concluded at the time that the rate of day-case surgery in Scotland could be substantially increased [40]. The number of day surgery and outpatient procedures were set as key national performance indicators as part of the NHS strategic health policy priorities HEAT (Health, Efficiency, Access and Treatment) programme, with a target to achieve 80% of BADS surgical procedures performed in a day case or outpatient setting by March 2011 [41, 42].

Table 1 present the number and ratio of inpatient vs. day case elective BADS procedures in NHS GGC, over 5 years, from 2007 until 2012. This time frame coincides with the PCIP review of PACs in NHS GGC (2008) and the roll-out of the POA eForm (2009). The figures were obtained from the Operations and Procedures - Hospital Care data set published in September 2012 by the Information Services Division, ISD Scotland (the statistical services of NHS Scotland)^b^. ISD has reported no known data issues for inpatient/day-case figures. However, it has highlighted that recording levels have increased since October 2011 for outpatient procedures. Thus, the standardisation of POA processes and the roll-out of the eForm should be seen in the context of concerted efforts within NHS GGC to improve the efficiency of the surgical pathway in the health-board over this period.

**Table 1:** **Elective BADS procedures, inpatients, day-cases and outpatients NHS GCC Health-Board,** ***(source: ISD, Operations and Procedures - Hospital Care, Sept. 2012)***

Outcome indicator	2007/08	2008/09	2009/10	2010/11 ^∗^	2011/12 ^∗^
Elective BADS procedures	53,890	56,101	57,666	69,874	76,326
BADS procedures as inpatient	16,908	17,278	15,802	15,171	14,373
BADS procedures as day cases	32,394	34,050	35,948	36,504	37,389
BADS procedures as outpatients	4,588	4,773	5,916	18,199	24,564
Percentage BADS procedures as day cases or outpatients ^∗^	68.6%	69.2%	72.6%	78.3%	81.2%
Percentage BADS procedures as day cases, excl. outpatients ^∗^	65.7%	66.3%	69.5%	70.6%	72.2%

^*^NOTE: ISD remark on data quality/completeness: ‘*outpatient figures recording levels have increased since October 2011*’.

## Case-study of POA eForm use in the surgical pre-assessment clinic

We conducted a case-study at the PAC of a general teaching hospital situated in the city of Glasgow, providing a broad range of medical and surgical sub-specialties, including day-surgery and inpatient surgery. The hospital has a number of inpatient wards for surgery, including ophthalmology / eye surgery, orthopaedics, general and vascular surgery, Ear, Nose and Throat (ENT) surgery, gastrointestinal surgery, urology as well as a high dependency surgical ward. The PACs provide a generic POA pathway for general surgery and other surgical specialties:

- **Nurse 1**:*“[...] The pre-op assessment [...] is a generic pre-op assessment which means we pre-op assess for several specialties; so, just for example we do gynaecology, general surgery, vascular, ENT. The pre-op assessment is basically a general health check prior to this patient being scheduled for surgery, just to make sure that they’re fit for that surgery and if there is any risk identified that the risks are managed so that they can safely get their surgery."*

**PAC appointment service model:**

The PAC operates a ‘walk-in’ clinic model, which means that – if the surgeon has made a preliminary decision to operate pending the outcome of the POA review – a patient will present for appointment at the PAC immediately following on from a consultation at the outpatient clinic. The PAC aims to provide a ‘one-stop clinic’ service, meaning that investigations should be carried out in a single visit, unless the patient requires further investigations or referrals (e.g. to cardiology) which can not be accommodated on the day. There are other referral routes to the PACs (e.g. from other hospitals) and at the time of the interview, a standardised referral process was planned – but not yet in operation – by setting up a dedicated electronic mailing list which would be examined by the POA nurses twice a day.

For patients who need a booked appointment to the PAC, it was reported that an appointment would usually be available within 2 to 3 weeks from an initial referral to the PAC. Each nurse will usually be allocated 8 patient appointment per day, although this figure naturally varies according to clinical requirements, such as emergency appointments. Each nurse has her own patient appointment folder in the electronic portal so that she will know exactly the list of patients she will see on anyone day.

**The patient preoperative interview:**

The clinical portal allows the nurses to have access to all available previous patient medical history before proceeding to their own assessment. They then proceed with the structured questionnaire, with conditional branching and embedded guidelines for further investigations (see Additional file 2: Appendix-8). The nurses can also provide additional comments as free text under certain questions. Nurses also provide information on the patient’s current treatment and medication, including dosing and frequency.

**Protocol-based pre-assessment:**

An important implication of the fact that the guidelines are now embedded in the electronic ICP means that the nurses no longer need to refer to a multitude of external resources as they used to do when completing a paper-based POA questionnaire. This naturally facilitates the flow of the patient assessment, as well as ensuring that all nurses work to agreed standards. The ICP thus has become an important tool in promoting the adherence to strict guidelines during POA across all the sites in NHS GGC (see Additional file 2: Appendix-9).

**Management of screening tests and investigations**

Screening tests are routinely used as part of POA either to uncover undiagnosed conditions or investigate the severity of known conditions. However, the evidence to manage the ordering and interpretation of these tests remains elusive. Previous systematic reviews on the use of screening tests during POA found a lack of evidence underpinning the effectiveness of routine testing and a lack of discernible impact of patient management practices [17, 43]. The ICP is used as a screening management tool: for ordering tests based on the agreed nursing guidelines, to follow-up on test results and coordinate action as necessary and potentially as well as an auditing tool for resource utilisation (see Additional file 2: Appendix-10).

**Anaesthetic or specialist medical review**

The PAC is nurse-led and the standard clinical pathway would be for the nurse to conduct an assessment with a patient without any further medical input, unless support is requested for the more complex patients. There are no dedicated anaesthetists allocated to the PACs and their input is on a case-per-case basis, when the consultants are contacted by the nurses as needed. The anaesthetists will only have a direct consultation with the patient in exceptional cases. Occasionally for higher-risks patients, a surgeon may request directly that an anaesthetist review the patient’s notes before making a clinical decision on the patient’s suitability for elective surgery.

- **Interviewer:** “Do you see the patient sometimes?"

**Anaesthetist 1**:*“ Yes, I mean occasionally, I’ve got to see patients but that would only be... I don’t personally think there’s a huge benefit by me seeing patients beforehand because a lot of it can be done by discussing with the nurses, and it’s only if you come to a situation where you’re not sure if the patient’s really fit for the surgery, you need to see them. That’s my personal view, some people are keen to see them in the clinics because they think they’ve seen them beforehand and discussed it, but..."*

If a nurse has any cause for concerns regarding a specific patient, she can send an electronic message to one of the anaesthetists or another consultant (see Additional file 2: Appendix-11). An anaesthetic review request will appear in the consultant’s portal in-box and he can then access the patient’s preoperative summary, which highlights the patient’s nursing review, usually relating to morbidity history and events. In most cases, the anaesthetist will conduct his review remotely using the documentation available on the portal.

**Managing surgical admissions**

The aims of POA is not only to assess the risk of surgery but also to allow effective patient planning. This will naturally include managing the patient admission route into hospital (i.e. day-surgery vs. inpatient). The ‘High Impact Changes for Service Improvement and Delivery’ report recommended establishing day-case surgery as the preferred hospital admission route for all eligible patients [44]. The nurses record on the clinical portal their recommendations for a preferred admission route based on their assessment of the patients (see Additional file 2: Appendix-12). Improved patient admission management in turns bring benefits in term of increased day-cased admissions and fewer cancelations on the day of surgery.

- **Anaesthetist 1**:*“[...] I think the benefits which will... are starting to happen at... – which would happen if there’s probably more investment resources throughout the hospitals –...that patients are fully prepared now for surgery, their... All the correct information allows for better scheduling ’cause we now know, you know... Patients who are admitted generally won’t be cancelled because of poor preparation. It also means that patients are now able to just come in on the day of surgery. You could argue that there will be few people admitted the day before surgery... So that sorts bed occupancy issues... and that’s the main sort of efficiency [...] If someone’s cancelled now [...] that will be reported and it will be looked into. Very few people are now cancelled on the day of surgery, because of poor preparation."*

**Perceived benefits & dis-benefits of the POA electronic integrated care pathway**

It is important to highlight that paper-case-notes are still being used throughout the patient pathway in the hospital. The nurses will perform their POA, then print a copy and file it in the patient paper record. In part this is due to the fact that the nurses in the surgical wards do not have easy access to computers and therefore still need to rely on using paper copies of the patients’ medical records. Also some consultants still insist on using the paper case-notes, although they may have easy access to the portal. The management of paper-based records is obviously a considerable logistic issue throughout the NHS as we have highlighted in previous studies of primary care electronic records and electronic transfer of referrals [6, 8]. One recurrent problem is when the record is simply not physically accessible, for a variety of reasons: either being used by a different member of staff (e.g. a consultant), being in a different location in the hospital, in a different hospital or even in another health-board (see Additional file 2: Appendix-13). The latter is not uncommon as many patients from other health-boards are routinely referred to NHS GGC ACHs for specialist surgery. Within NHS GGC, accessing the notes through the clinical portal goes some way in resolving these logistic issues. However, accessing information from other health-boards can remain an issue.

The nursing staff considered that the main advantage of using an electronic integrated care pathway was to have a central, accessible repository of information which allowed effective information sharing among the multi-disciplinary team (see Additional file 2: Appendix-14). This echoed the opinions expressed at other PACs in NHSScotland which had implemented electronic POA ICPs [1].

Few dis-benefits associated with the POA eForm were highlighted by the nursing staff and these were mostly associated with common problems with any IT system, such as occasional system breakdowns. The nurses felt that the system could also be slow to respond and all 3 POA nurses interviewed felt that completing the electronic ICP could take longer than the paper version (see Additional file 2: Appendix-15). One nurse highlighted that she felt she lacked IT literacy and was initially reticent to use the eForm but finally got round to using it more comfortably.

- **POA Nurse 4**: *“Well at first I didn’t really like it, I must admit ’cause I’m... I may be old-fashioned I think... I’m not... well I’m faster than I was, but I wasn’t very fast at typing initially and I find the writing a lot quicker. But I got used to it and now I find it better"*

One nurse mentioned that it affected the amount of eye-contact she made during the consultation. This impact of computer use on the patient-clinician encounter during the consultation has been highlighted in other studies but was not necessarily associated with a decreased satisfaction from patients, as these tend to show an acceptance that IT systems are becoming an integral part of part of the consultation process [45, 46].

- **POA Nurse 2**: *“... the only thing I find is you’re not making as much eye-contact with the patient when we’re actually using the computer because you have to sit and although you’re kind of looking up every now and then, with the paper copy you would sit-in addressing the patient, you were sitting face-to-face with them. There were more eye contact [...] I think it’s just me that I’m thinking like that, I’m thinking maybe I’m not making as much eye contact as I should with this patient..."*

Finally, it is also worth pointing that as gradually more and more systems gets integrated within the electronic portal, more information is becoming accessible to the nurses. However, this is a work-in-progress and the nurses will still need to chase up bits-and-pieces of information during the POA.

- **POA Nurse 3**: *“The most part of when it’s working, it’s great: we love it, ’cause everything’s right there. There are some kind of shortfalls and some information gaps in the information... but just ’cause it’s not on there. But anything that’s on there is really easier to access. It’s really easy to access our documents. Upstairs can access our documents if for one reason or another it’s been lost in the patient’s file. I mean... when it’s working, it’s great! You have computer issues obviously... kind of lost without it. You know what I mean..."*

**Implications for patient management across the health-board**

As has been highlighted previously, NHS GGC PACs were substantially reorganised as part of the PCIP and therefore, the implementation of the integrated care pathway on the clinical portal needs to be seen within the context of a comprehensive reorganisation of services, which saw many changes to work practices over the last 5 years. Overall, the nurses interviewed felt that this resulted in a more streamlined and efficient service (see Additional file 2: Appendix-16). The patient POA ICP is accessible to all hospitals within the health-board, which has important implications for clinical practices. Patients are normally pre-assessed in the hospital where they have been referred to by their primary care practitioners. However, they will be seen by consultants who will routinely be working across several ACHs. Once a consultant has made a decision to operate a patient, the patient may have his surgery in a different hospital within the health-board, depending on the surgical specialty and the location of the surgeon’s operating list. One nurse highlighted that the portal was also particularly useful when other services contacted them, asking for specific information regarding individual patient case-management. The portal was thus found to facilitate continuity of care and this benefit was also highlighted in our study of primary care electronic records [6].

Finally, one of the nurses felt that an increasing amount of their time was being taken by administrative duties carried out on behalf of other services within the hospital (i.e. the pre-admission unit or surgical wards). Although this may be a symptom of greater integration between services, it also placed additional administrative burden on the PAC staff.

**Areas for service improvement**

The nurses also suggested some possible improvements to certain aspects of the service: communications with other departments within the hospital (the pre-admission service, the surgical wards), quicker feed-back from (some of) the anaesthetists following review requests, improved (paper) case-notes management throughout the hospital, further online access to some screening investigations, and clearer protocols for managing certain categories of patients (e.g. substance users). One nurse felt that other services within the hospital were not always fully aware of the work that was performed at the PAC and that this could lead to inappropriate expectations or referrals. For example, some surgical consultants were sending patients to the PAC for a medical examination by a doctor or an anaesthetist while the PAC is nurse-led and without routine access to doctors.

## Discussion

Our results on the study of the design and deployment of the POA eform in NHS GGC suggest that this electronic health implementation has been broadly successful and that the electronic portal is now embedded in clinical practices. The number of regular users of the system (over 180 nurses and anaesthetists across the health-board) and routine assessment now performed (over 700 weekly assessment in January 2013) indicate that the use of the POA eForm is now normalised in routine assessment, both for preoperative nurses and anaesthetist consultants. Our study set to identify the factors which facilitated this successful implementation in order to instruct future large scale eHealth implementation in this sphere. Clear synergies between the parallel efforts of the planned care improvement programme and the eHealth programme have both contributed to the standardisation of preoperative documentation, guidelines and integrated care pathways which have then allowed the new preoperative clinics developed as part of the PCIP in NHS GGC to operate using the same electronic information system, under coherent clinical processes. These synergies are illustrated in Figure 5.

- to start with, there was an obvious and clearly identified clinical need for streamlining the elective patient pathway across NHS GGC. Prior to the PCIP, preoperative assessment practices across the health-board were fragmented, ad-hoc and inconsistent. Many hospitals had basic and surgical-specific pre-assessment procedures; others, none at all. This situation was indeed not suitable for operating modern and coherent integrated elective surgery pathways across the health-board.
- The impetus for the redesign of elective patient pathways was thus provided by the PCIP. The programme oversaw the rationalisation and streamlining of services and the development of new dedicated preoperative clinics, servicing the whole elective surgery population at a hospital instead of each services operating in silos.
- In addition, the HEAT target for BADS procedure – requiring that all NHS health-boards reached a measurable efficiency target by March 2011 of 80% of BADS procedures as day-cases or outpatients – provided an immediate incentive for NHS health-board and hospital managers to operationalise new POA clinics and elective pathways without delay.
- The NHS GGC electronic patient record programme provided resources to streamline and standardise POA documentation and guidelines across the health-board. In doing so, it not only consulted widely with a range of stakeholders across the health-board, but also ensured that the implementation was clinically-led by a senior anaesthetic consultant in order to lead the POA guideline and protocol standardisation and the system clinical decision support functionalities. Furthermore, the EPR also liaised closely with the POA nursing staff throughout the iterative phases of the POA eForm design, development, testing and implementation.

Previous reviews of the literature on preoperative assessment processes in elective surgery have suggested that the standardising of POA processes and data collection methods is extremely difficult to achieve in practice [47–49]. The complexity of the domain – with multiple disease or surgery specific-protocols on the one hand and the lack of guidance for the management of complex multi-morbid patients of the other – has led to the operationalisation of data collection instruments and assessment protocols which often depend on local clinical policies and priorities, rather than robust evidence of effectiveness [35, 50]. In 2007, the NHS Connecting for Health programme in England performed a broad consultation with stakeholders leading to the development of a standard POA dataset, but to the best of our knowledge, we are unaware of practical implementations currently using this dataset. This project was later discontinued and is no longer supported. Ahmadian et al. performed a systematic review to identify a core data set of information routinely gathered during POA but concluded that due to the diversity of clinical goals, a standardised data collection tool (e.g. patient questionnaire) may not necessarily be feasible. However, they recommended the adoption of peer-accepted standard reference data sets, in order to facilitate meaningful comparison of services and data-sharing among multiple health-services providers [51]. The successful standardisation of POA data collection processes across NHS GGC is therefore a substantial achievement.

Using the 4 NPT constructs, we review and interpret the findings of our study.

**• Coherence:**

Coherence refers to the “sense-making" work undertaken when a new e-health service is implemented: to determine whether users see it as differing from existing practice, have a shared view of its purpose, understand how it will affect them personally and grasp its potential benefits [5].

It is clear that considerable effort was put into policy building and dissemination of information to all stakeholders across NHS GGC.

The impetus for whole service redesign in the health-board came from 3 national programmes: the PCIP, the eHealth clinical portal programme, and the HEAT target for BADS procedure.

As the stakeholders we interviewed have explicitly stated during the interviews, the rationale for PAC services re-design was not only well-understood but also actively supported. The traditional surgical admission route as inpatient, with minimal prior anaesthetic review, was leading to well-know identified clinical and workflow issues within the patient pathway: last minute medical history elucidation and screening, insufficient patient preparation, late surgery cancelation and possibly delayed discharge. Importantly, while the need for PAC services redesign – driven by the PCIP – was evident for stakeholders on the ground, the two other programmes both provided the tools (eHealth clinical portal POA eForm) and the impetus (HEAT target) for changes to be enacted in practice.

The rationalisation of guidelines across the health-board was again justified by the fact that the absence of a coherent framework was leading to practical difficulties for patients and the MDT alike. We have highlighted that consultants often work across hospitals in the health-board and patients can be pre-assessed in a hospital only for their surgical procedures to take place in another ACH. Nurse 1 stated that this had led to instances of patients being cleared for surgery at one site only for them to be refused surgery at another due to differing nursing criteria being used at either sites. With the guidelines having now been agreed across the health-board, with a standard POA ICP in use and accessible from all ACHs and much greater scrutiny of the reasons behind surgical cancelations, the risk of contradictory decisions regarding patient management based on arbitrary variations in pre-admission protocols appears to have receded.

Nurse 1 also played a role in disseminating information across the health-board throughout the PCIP. These activities clearly suggest that a considerable effort was made to engage with relevant stakeholders and promote a *coherent* rationale for service redesign.

**• Cognitive participation:**

Cognitive participation focuses upon the work undertaken to engage with potential users and get them to “buy into" a new e-health system [5]. The work of relating and engaging with users is central to the successful implementation of any new technology.

While consultants were the main driving force behind the development of common guidelines, the POA eForm could not have been operationalised in practice without the active support of the nursing staff, since they conduct the bulk of the pre-assessment work – as a nurse quite rightly emphasised: (*‘we’re the one using every day...’*) The EPR programme close involvement of the nursing staff throughout the design and user-testing of the eForm was hence a critical factor in that respect. The nursing staff felt that they had been consulted, that their advice was heeded and that system changes were operationalised in a timely manner. They appreciated the fact that they had been active contributors in ‘shaping’ a tool that could suit their work practices and protocols. This was an important factor in legitimising the implementation of the POA eForm and clearly facilitated its rapid deployment and adoption within routine practice. In addition, the close involvement of Nurse 1 – both in the PCIP review and POA eForm user-interface design and testing – meant that the ICP roll-out immediately benefited of the presence of a local *champion* in NHS GGC.

The eForm IT team was responsive to the nursing needs throughout the design and implementation of the ICP and equally, the PAC nursing staff felt they could clearly communicate design and change requirements to the eForm team. A perfect example of how these design recommendations translated into a practical implementation is the short day-case / outpatient first-line ICP.

**• Collective action:**

The emphasis of collective action involves the work performed by individuals, groups of professionals or organisations in operationalising a new technology in practice and sociotechnical issues, such as how e-health systems affected the everyday work of individuals and organizational structures [5]. The impacts of the POA ICP in that respect are substantial and overall it is clear that the uptake, adoption and normalisation of the eForm has been possible because, to a large extent, it has made the completion of clinical tasks during assessment easier.

Embedding the guidelines within the electronic ICP has enabled the standardisation of nursing assessment criteria across the health-board. Decision support at the point of care in the form of adaptive conditional branching and advice throughout the patient interview means that protocols are clearly defined and adhered to, without the need to refer to external documentation and guidance. Naturally, the guidelines can never be entirely exhaustive and nurses will still need to rely on their experiences and judgement throughout the patient assessment. The clinical portal can be an extremely useful tool in that respect due to the comprehensive information that can be accessed through the system: clinical notes, referrals, investigations, X-rays and so on. It means that the nursing staff can make *informed* decisions about individual patient case management based on a comprehensive medical history. In addition, this information can be effectively reviewed and shared by the members of the MDT. This aspect of the PAC is essential as a previous review has suggested that effective communication and information sharing across the perioperative pathway is essential for the delivery of safe outcomes for surgical patients [52].

*Roles, responsibilities and training:*

Nursing staff at the PAC were highly experienced in the care and management of surgical patients. All staff received in-house competency training as well as training in the use of the POA ICP. One potential issue however was the lack of formal arrangement for medical support at the PAC for complex patients. The nursing staff can request anaesthetic reviews of patients case-notes from the consultants via the eForm but this is usually performed remotely, through the consultants assessing the patient’s medical history via the clinical portal. Anaesthetist 1 suggested that it was unusual for consultants to perform physical examinations of patients prior to the day of surgery and this was in stark contrast to some other PACs in Scotland, which had weekly dedicated anaesthetist-led clinics to support the nursing staff in the assessment of the more complex patients [1]. Some nurses suggested that getting timely feed-back from the anaesthetists could be difficult at time. There is currently a lack of robust evidence on the impact of nurse-led pre-assessment clinics on surgical outcomes [53, 54]. Equally, the lack of clear outcomes indicators for surgery within NHSScotland in terms of patient cancelations and adverse events does not permit to make reasonable comparisons between distinct services models (i.e. with or without anaesthetist-led clinics).

**• Reflexive monitoring:**

Reflexive monitoring deals with the evaluation and monitoring of eHealth implementations and how these are used to influence utilisation and future implementations [5]. Much of this was in evidence throughout the POA ICP implementation.

The eForm was developed following extensive consultation with the nurse through an iterative, user-centred, design process and it the POA nurses emphasised that this was an on-going process, i.e.: that the design process is not closed and remains active throughout the implementation. Change requirements and requests can be communicated to the EPR eForm team on an ‘as-needed’ basis and these will be incorporated into future iterative versions. The fact that the system is actively supported is important to ensure that the system remains technologically up-to-date as well as continuously relevant to nursing practices and processes. We have witnessed elsewhere how the technology underpinning a POA ICP can within the space of a few years become obsolete [1].

In addition, the eHealth clinical portal and eForm programme have regularly held information dissemination workshops during which NHS staff could both share their experience of these implementations or learn how these systems were operationalised in practice. Interestingly, the focus group which took place in August 2011 between the NHS Tayside nursing staff and the NHS GGC POA and eForm team subsequently led to the deployment of the clinical portal in the PAC of a NHS Tayside ACH the following year. This demonstrates that the experience gained in the implementation of these systems has not only been actively shared within NHS GGC but with other health-boards as well and nationally, through the activities of the eHealth programme.

**• The sociotechnical systems approach to eHealth implementation:**

A recent study by Bardhan & Thoin on the impact of ICT on the quality of healthcare delivery highlighted that the usage of healthcare IT solutions could have a positive impact on clinical processes, compliance to evidence-based guidelines and quality of care [55]. From the perspectives of sociotechnical systems, it is clear that implementing and embedding new technologies involves complex processes of change both for health professionals and patients at the local level and for the organisation of health services [5]. It has long been recognised that these changes can cause serious disruption to services, potentially leading to ‘unintended consequences’ once new technologies are deployed in context [56, 57]. Furthermore, evaluating the impact of these changes also present substantial methodological challenges, requiring a ‘deep understanding’ of the context and complexity of the problems that eHealth interventions attempt to address [57–59].

Maguire has suggested that for a sociotechnical systems approach to be effective, the following fundamental considerations are essential: (i) having realistic expectations about the system development and a grasp of the potential complexity of the tasks that a new system will require from users, (ii) having realistic expectations about the impact of the new system on work processes, (iii) allowing for some flexibility of systems functionalities in order to address preliminary feedback from users, (iv) having a flexible software environment which allows for some adaptation to users’ needs and finally, (v) involving users early on, so that they can specify the benefits that they expect from the new system [60]. Our results suggest that the design and development of the POA eForm met most of those fundamental considerations. Specifically: the early involvement of health professionals and preoperative nurses in the functionalities development and iterative testing phases almost certainly significantly contributed to the successful deployment. As an example, at the other end of the implementation spectrum, the study of 4 EHR implementation case-studies by Eason & Waterson concluded that strategic and management needs often took precedence over front-line staff, who were consulted only once the systems were implemented [61]. One of the major drawback of such a *‘top-down’* approach to implementation was that the information needs of health professionals were often not adequately met, resulting in ad-hoc work-around such as data-entry tasks duplication in a variety of information systems. In a recent analysis of 3 implementation cases-studies within the National Programme for Information Technology, (NPfIT), Waterson highlighted the tensions which often existed between the national policy and local clinical priorities [62]. A clear opportunity was missed when the dire needs for electronic solutions (i.e. electronic health records, virtual wards and electronic portals) to support clinical tasks, care coordination and integrated care pathways were ultimately not met, as systems were not perceived to be *fit-for-purpose* by clinical staff. The lack of interoperability of diverse IT systems within the patient pathways often became an insurmountable issue for integration. Ad-hoc work-around developed as a result of this lack of system integration could be considered to increase the risk of information or decision-related errors and potentially be detrimental to patient safety. In one of the three case studies (Stroke Pathways), the IT support for clinical tasks was fragmented and conflicted to some extent with the community-care team clinical practices [62]. Waterson further suggests that the key lesson to emerge for future eHealth system deployment is that using a sociotechnical systems approach to implementation, including consulting with clinical staff prior to designing eHealth systems in order to capitalise on the potential enthusiasm for new electronic solutions and conducting a thorough mapping of work-flows and task-related roles are among some of the essential requirements to deploying fit-for-purpose eHealth systems. This is also the approach recommended by Harrison et al. in their recursive model of Interactive Sociotechnical Analysis (ISTA), which advocates the inclusion of regular feedback loops in a system implementation in order to allow for ‘second-level’ social adaptation to maximise the chances of a system being deployed successfully. Our study results certainly support these recommendations.

**• Study strengths and limitations:**

The main study limitation is the small number of participants which we interviewed. However, we also attended a focus group with members of the POA MDTs of NHS GGC and NHS Tayside and members of the EPR programme as well as 2 workshops organised by the EPR programme. We have collected a wealth of qualitative data as should be self-apparent in this study. One of the study strengths is that we succeeded in interviewing key architects of the POA eForm implementation and stakeholders in both the Planned Care Improvement programme and Electronic Patient Record/Clinical portal programme.

It would be useful in future to conduct a broader survey on a larger sample of users of the POA eForm in order to ascertain perspectives on the usability of the system, match to work practices and how it may have impacted clinical processes and patient care.

## Conclusion

This study describes the policy context and analysed the sociotechnical factors which have led to the successful implementation of standard preoperative processes across the PACs of NHS GGC and allowed the deployment of an electronic integrated care pathway, underpinned by consensus guidelines.

Key factors which contributed to the successful rationalisation of preoperative clinics and the deployment of the electronic integrated care pathway include:

(i) *a strong national policy context for the rationalisation of processes and data collection instruments for surgical pre-assessment, including efficiency indicator targets for specific procedures (i.e. the HEAT target of ratio of day-cases and outpatients vs. inpatients for BADS procedures),*
(ii) *financial and organisational resources to support the review and redesign of preoperative clinics and the operationalisation of standard electronic information collection and sharing systems*
(iii)
*a sustained engagement with preoperative nurses and consultants throughout the iterative phases of preoperative clinics development, guideline and documentation standardisation and the eForm design and implementation,*
(iv)
*the use of a pragmatic and domain-agnostic technology solution,*
(v)
*finally: a consensual and context-specific implementation, based on national guidelines as well as local clinical expertise and protocols*

## Endnotes

^a^ Scottish Parliament, Health and Sport Committee Report HC/S3/10/R3 http://archive.scottish.parliament.uk/s3/committees/hs/reports-10/her10-03.htm

^b^ ‘Annualṫrendsi̇nḂADSṗroceduresḣbtṠep12.xls’ available at http://www.isdscotland.org/Health-Topics/Hospital-Care/Operations-and-Procedures/

## Electronic supplementary material

Additional file 1: **Qualitative RATS Check-List’.** ANNEX I - qualitative RATS check-list integrated preoperative care pathway - A study of a regional electronic implementation. (PDF 39 KB)

Additional file 2: **Appendix - Additional quotations from the study participants.** (PDF 63 KB)

## Abbreviations

ACH: Acute care hospital
BADS: British Association of Day Surgery
CHI: Community health index
ECS: Emergency care summary
eForm: Electronic form (also: electronic Integrated Care Pathway in the context of this article)
ENT: Ear, nose and throat
EPR: Electronic patient record
GP: General practitioner
ICP: Integrated care pathway
ICT: Information & communication technology
MDT: (preoperative) Multi-disciplinary team
NHS GGC: NHS Greater Glasgow and Clyde health-board NHSScotland: National Health Service for Scotland
NPfIT: National Programme for Information Technology
NPT: Normalisation process theory
OT: Operating theatre
PAC: (Surgical) Pre-assessment clinic
PCIP: Planned care improvement programme
POA: Pre-operative assessment
SCI: Scottish care information group
SCI Gateway: The national eReferral system developed by the NHSScotland SCI group
SCI Store: Electronic data repositories managed by each of the regional health-boards.
