# Supplementary material for: Implementation of an integrated preoperative care pathway and regional electronic clinical portal for preoperative assessment
Source: BMC Med Inform Decis Mak. 2014 Nov 19;14:93. doi: 10.1186/1472-6947-14-93 (PMC4289251; doi:10.1186/1472-6947-14-93)
Supplement: Supplementary file 1 — Additional file 1: Qualitative RATS Check-List’. ANNEX I - qualitative RATS check-list integrated preoperative care pathway - A study of a regional electronic implementation. (PDF 39 KB) [file 12911_2013_876_MOESM1_ESM.pdf]

BMC Medical Informatics & Decision Making

Implementation of an Integrated Preoperative Care Pathway and Regional Electronic Clinical Portal for Preoperative Assessment

ANNEX I - Qualitative RATS Check-List

Matt-Mouley Bouamrane & Frances S. Mair

I- Research Questions:

- *What were the key sociotechnical factors which contributed to the successful implementation of elective surgery integrated care pathways and the preoperative electronic portal (eForm) in the NHS Greater Glasgow and Clyde (GGC) health-board?*
- *What were the impact and potential synergies of national policies on these implementations: including: the Planned Care Improvement Programme (PCIP), the Electronic Patient Record / Clinical Portal programmes and the Health, Efficiency, Access and Treatment (HEAT) British Association of Day Surgery (BADS) targets?*
- *What were the perspectives of end-users of the preoperative eForm in one selected case-study in an Acute Care Hospital (ACH) in NHS GGC?*

II- Qualitative Method:

- *Semi-structured interviews (n=3) with stakeholders in the preoperative electronic portal (eForm) implementation in NHS GGC.*
- *Semi-structured interviews (n=3) with nursing staff routinely using the preoperative eForm during assessment (Surgical Pre-Assessment Clinic of ACH in NHS GGC)*

• *One focus group with preoperative multi-disciplinary teams from NHS GGC and from NHS Tayside and members of NHS GGC Electronic Patient record programme*

• *Field notes from 2 workshops organised by NHS GGC Electronic Patient record programme*

III- Sampling / Recruitment / Data Collection:

Contacts were made with NHS GGC via email in order to identify the people involved in the development and implementation of the electronic preoperative clinical portal. Three stakeholders were identified and contacted by email. We provided background information on the purpose of this study and suggested arranging a date for an interview. All the three stakeholders approached agreed to take part in an interview. These were:

- **eForm 1:** a member of the NHS GGC electronic patient record programme (EPR) eForm team involved in the development of design requirements and technical specifications for the preoperative ICP,

- **Anaesthetist 1:** a consultant anaesthetist involved in the consensus process which led to development of the structured clinical content of the preoperative ICP, including the selection of guidelines underpinning the context-dependant, adaptive behaviour of the eForm.

- **POA nurse 1:** a senior nurse involved in the PCIP review of the NHS GGC PACs and the dissemination of information relating to the programme implementation across the health-board. In addition, the nurse was involved in the eForm user-testing, reporting user requirements and change requests to the eForm development team.

In addition, to these 3 interviews, we also conducted in February 2012 a case-study at one preoperative clinic in an NHS GGC Acute Care Hospital (ACH). On that occasion, we interviewed the service lead nurse and 3 nurses who worked in the clinic. The nurses were routine users of the preoperative eForm during patient assessment.

Interviews duration ranged from over 20 minutes to over an hour and 20 minutes, with a mean duration of approximately 43.5 minutes per interview. The interviews were semi-structured and open-ended in order to allow the interviewer or interviewee to elaborate on unanticipated and

potentially valuable information with additional questions, and probe for further explanation

• In addition to the above interviews, a focus group organised by the NHS GGC POA team and members of the EPR eForm programme took place in August 2011 in one of the NHS GGC ACHs. The aim of this meeting was to present the implementation of the electronic portal and POA eForm to a nursing, IT and clinical management delegation from NHS Tayside. A researcher (M-M.B) was invited to attend the meeting. The other participants in this meetings included 2 members of the NHS GGC EPR eForm project and – from NHS Tayside – a nursing manager, 2 POA nurses and a member of the ACH IT department. The meeting duration was just slightly under 3 hours and was digitally audio-recorded by the researcher with the explicit consent of all participants.

• Finally, one researcher (M-M.B.) was invited to attend 2 forums organised by the NHS GGC EPR programme. These workshops lasted for a full-day and aimed to provide a platform to share experiences on a range of eHealth implementations across NHSScotland. Participants were members of the eHealth programme and NHS staff from various health-boards. This was an opportunity for the researcher to take notes and discuss the stages of implementation of the clinical portal in NHS GGC and other health-boards with a range of active stakeholders.

IV- Data Analysis:

• *Over 7 hours and 15 minutes of audio recording were transcribed verbatim and qualitatively analysed.*

• *We used process-mapping techniques to model POA processes in NHS Greater Glasgow and Clyde.*

• *We then used Normalisation Process Theory (NPT) as a conceptual framework to interpret the factors which were identified as facilitating or hindering the work of the members of the preoperative multidisciplinary (MDT). NPT is concerned with the social organisation of the work (implementation) of making practices routine elements of everyday life (embedding) and of sustaining embedded practices in their social contexts (integration) and was developed particularly in response to the evidence, which suggested that electronic health implementation, embedding and integration*

are difficult to achieve in practice.

- *The interview transcripts were analysed and coded by one researcher (M-MB). The two co-investigators (MML & FSM) then discussed the coding framework used on the transcripts in "coding clinics" to ensure a consistent approach to coding and the validity and robustness of the proposed coding framework.*

This thematic framework was designed on the four key generative mechanisms of NPT: coherence; cognitive participation; collective action and reflexive monitoring.

- **Coherence:** *refers to the work of making a complex intervention hold together and cohere to its context, how people "make sense" or not of the new ways of working.*
- **Cognitive participation:** *is the work of engaging and legitimising a complex intervention, exploring whether participants buy into and/or sustain the intervention.*
- **Collective action:** *examines how innovations help or hinder professionals in performing various aspects of their work, issues of resource allocation, infrastructure and policy, how workload and training needs are affected and how the new practices affect confidence in the safety or security of new ways of working.*
- **Reflexive monitoring:** *is the work of understanding and evaluating a complex intervention in practice, and how individuals or groups come to decide whether the new ways of working are worth sustaining.*
