# Supplementary material for: Implementation of an integrated preoperative care pathway and regional electronic clinical portal for preoperative assessment
Source: BMC Med Inform Decis Mak. 2014 Nov 19;14:93. doi: 10.1186/1472-6947-14-93 (PMC4289251; doi:10.1186/1472-6947-14-93)
Supplement: Supplementary file 2 — Additional file 2: Appendix - Additional quotations from the study participants. (PDF 63 KB) [file 12911_2013_876_MOESM2_ESM.pdf]

BMC Medical Informatics & Decision Making

Implementation of an Integrated Preoperative Care Pathway and Regional Electronic Clinical Portal for Preoperative Assessment

ANNEX II - Additional Quotations from the Study Participants

Matt-Mouley Bouamrane & Frances S. Mair

1. Aims of preoperative assessment:

- **Anaesthetist 1**: *"The benefits of preoperative assessment services now are that the patients are seen by a person – well... seen by a nurse – who can do a health-screening or a health-assessment. And this can then trigger a referral to other specialties when required or to anaesthetists. They can also provide more information to the patients. Information about the pathway of care, they can come and ask us that. Patients can also be given information about surgery – the benefits of surgery – (and) can be consented. All this happens, hopefully, in one visit to the hospital. It means that when a patient is admitted to hospital and they do a surgery, there shouldn't be anything else that needs to be done. Everything should be completed so that when the anaesthetist eventually see the patient, they go through it all and they'll be nothing really that will prohibit surgery from proceeding on that day or the following day. Systems before that: you'd have a patient who would come to hospital, go to see a surgeon, booked-in for surgery then come to hospital, the day before the surgery having had not other intervention. And your patients turned up, who weren't properly optimised for surgery, didn't have any investigations carried out, wouldn't have had any other nursing administration carried out... So there's a lot of work on the day, immediately prior to surgery. They would maybe face having to go for a barrage of investigations at the very last minute, to*

drag it down. Or they may even be faced with a cancellation 'cause they weren't ready to go for surgery... so patients are now prepared better for surgery."

- **POA nurse 1** : *" Just clarify anything [...] it's for gathering as much information as you can so that you can make a proper decision on that patient, all... pre-op's all about is managing risk, identifying and managing risk more than anything..."*

2. Standard guidelines development across the health-board:

- **Anaesthetist 1**: *"[...] We developed our guidelines based upon best available evidence at the time but it's not been the same process that for example the NICE* (National Institute of Clinical Excellence) *guidelines would use. You know... we certainly used local opinion, local current practice which has been modified and we've used any national guidelines to help develop our own local interpretation of that. So our guidance – and it's been agreed throughout Greater Glasgow and Clyde – so at the time of development, the guidelines were set up and then they were sent to all the main users, all of the consultants who were... had expressed interest. Each individual then reviewed the guidelines, made comments, then we changed them. So it's... it's as consensual as it could be without doing a proper formal consensus process. [...] We did have workshops, the sister in pre-assessment at [ACH name]: she was involved with one of the managers [name] and they coordinated road shows at the time. They would go round to all the various pre-assessment units to introduce the guidelines and discuss them and then we had feedback. So it was done... yes, a lot of work went into it!"*

- **POA nurse 1** : *"[...] From the very start. It was part of... I was part of the pre-op assessment group which was: myself, an anaesthetist from each of the hospitals... each of the main hospitals. And then basically, what we did was, when we developed the guidelines, the guidelines went to every*

anaesthetist in Greater and Clyde and every health and community-health partnerships so it could go out to GP practices as well [...] We got a quite a bit of feedback but what I did as well, I went to every hospital and launched the document. So I had an open day for them, doing a presentation, showing them what it looked like and to get feedback from them as well. This was about setting up all these nursing guidelines; everybody having the same database so the information gathering was the same. And they're gathering it in the same ways."

3. Development of the NHS GCC clinical portal:

- **eForm 1**: *"[...] Greater, Glasgow and Clyde have been developing a clinical portal for a number of years. We are moving towards an... an electronic patient record, working paper-light. We will never work paper-less, because paper is always going to have a place. It's what we do with the outcome of that paper that we have to be considerate about: where we put it, where we store it, how we store it and what it actually contains? Is it clinical information? Does it have a place in the electronic patient record for other clinicians to see? Is it of benefit to them? [...] So when you've got a structured form and contents and you're capturing information electronically, it allows you to make sure you're... you're governing what goes into it. But that's been done with a lot of forethought because of engagement with people that want to see the information, and that goes without saying."*

- **Anaesthetist 1**: *"[...] We can get a lot of information off the clinical portal, all the letters are written by the medical specialists such as cardiologists or respiratory physicians will be in there. The X-ray result or any results of investigations: most of them are on the clinical portal."*

Interviewer: "And so: it's not just pre-assessment that is on it? Every thing... All the patient's record is on the..."

Anaesthetist 1: *"Not yet, not yet... but that what's the plan is... but any letter written to a GP or any correspondence coming from the hospital is on the clinical portal. All the notes I think [...] So it's building up... but any new letter typed or dictated, any new attendances at pre-assessment*

form will be electronically... Not all specialities em... are using the clinical portal yet, so I think it's something that's developing. We're one of the first to have access to it [...] All patients... If the surgeon... They write all patient notes, so that will be on. They'll be discharge letters so if the... you know... if the standard discharge letter goes to the GP, we'll be on [...] The only thing that aren't on are hand-written notes within the case-notes."

4. Portal integration with the national eHealth implementations:

- **eForm 1**: *"[...] this is a little bit about the... the infrastructure and the architecture around what we did. We had Greater Glasgow and Clyde SCI Store. We have a PAS* (i.e. Patient Administration System) *system in the south, a PAS system in the north and these are the... the patient administration systems where all your outpatients scheduling takes place, or your demographics are held here. We've also got SCI Gateway. Now, SCI Gateway; all the GPs will refer using that electronic SCI Gateway. But the clinical notes that are created in various systems... and these are stand-alone systems. We had radiology, where you got all your reports coming out of that department. You got all your laboratory results for all your procedures, investigations and you had letter systems. Now... We term these the 'feeder systems'. This is where all the information is created and generated: this is the starting point. We took all these systems and we integrated them into SCI Store. So SCI Store gets a copy of every piece of information that these feeder systems generate. SCI Store is never and will never be a primary data repository. It's a secondary data repository."*

- **eForm 1**: *"[...] When you file it in* (i.e. a clinical document) *electronically, there's standard indexing so that no matter what colour of paper, that referral – as it comes then – it's filed electronically in the same place. Think of all of your... all the hospitals that have got fluid balance-charts. Those fluid balance charts look different across every single ward, directorate, hospital, but it's still a fluid balance chart! So we needed to say: 'right, O.K... Regardless of what your flavours look like, they're all getting put in the same place, so they get filed in at the cabinet or fluid balance*

charts, then that's where they go electronically'. So that everyone knows where to go, where to look for something. So they can go in the clinical portal... put electronic information into that clinical portal without telling people where to go to look for it. So when you put information out and you say: 'right, there's now an e-form for pre-op assessment, you will find it in: clinical notes, other notes, outpatient record note, pre-op assessment document'. So you've got to tell people where to go."

5. Challenges of embedding decision support functionalities in the POA ICP:

- **Anaesthetist 1**: *"[...] That's why we limited the computer with the eForm, we thought 'well, you can have those guidelines working down to the background'. The main problem though with the guidelines with pre-operative assessment setting is: you have a patient who's got asthma, you know... it's really at a very – what do we call it...– moderate group of patients that, you know, if you've got a thousand patients with asthma, they're all very similar, so you can put down fairly straight-forward protocols or guidelines in how to... The problem with surgical patients are that no patients are the same. Every patient's different... You've got the surgical problem. Surgical problems may be varied to a huge amount, even in arthritis of the hip, it can be from straight-forward to difficult and then patients have got all sorts of different disease processes on-the-go. So you're trying to examine, assess, screen and then investigate these patients using guidelines and yet no one patient is the same. And I think that's a big problem with pre-assessing."*

6. Iterative user-testing:

Interviewer: "So of in terms of the clinical content of the form, was that you that designed that?"

- **POA nurse 1** : *"[...] No, that was the anaesthetists surely. And it was IT more than anything: there was a specific group of people who were brought in to develop... We were only specifically asked to user-test it and different things like that. And it took a lot of changes made to it, you know, because it wasn't until we started using it (that) we realised*

that there was gonna be other things missing and different things. So it's constantly changing and improving as we're going along."

- **POA nurse 2**: *"I think there's always room for improvement with it. I mean it's not a 100% fault-free at present, but it's getting better [...] We did have meetings last year where IT were on board and we were giving feedback regarding the site. So a lot of changes have been made since then as well though, which is really good. It was really good they wanted our input as users... 'cause we are the ones that using it everyday. [...] there's a lot that came out of it [...] Yes, definitely, they took our changes on board, which is good."*

7. ICP roll-out:

- **POA nurse 1** :*"We had a user-testing, then we went through it and then once we've said 'it's user-friendly', it rolled out pretty quickly [...] I sat and went through all of it and then I was to say 'that sort of things and that get to go through' and then they* (the eForm IT team) *did it [...] then we've got to what we thought was near enough what it was gonna be... that's what we went with. But they knew that if there were changes to be made they could still get made. And they still can, but there are a lot of electronic forms and not just the pre-op forms [...] we did a pilot of it and then looked to see then everybody's feedback. I mean some of the things I didn't... wasn't always me that identified that there was a problem somewhere. It was the nurses so I... Once we started using it, we were asked to gather and then look at it and then those changes [...] Yeah, I mean there's been lots of different things that have been added to it, and... [...] but at the end of the day, we were using it. It had to be quick so if there was changes to be made they had to be implemented kind of quickly but it's been like a trial and motion, kind of thing – if you know what I mean – because it's the first time we've ever had anything like that... But I do like it."*

8. The patient preoperative interview:

- **POA nurse 1** : *"So the first thing we'll get when we click the patient... we're gonna go and see is... that: we'll get this page which is clinical portal. From that, that will have his previous... if there's any previous documentation, any correspondence, just in case we don't have the case-notes. And it mentions medical lab results, but we'll go in through here and this will take us to the electronic e-form. [...] And then we'll go through... and we'll go through the general anaesthetic questions. So, if you can see there* (pointing on eForm on screen)*, if I'd had put 'no', there would have been the next question. Because I've said 'yes' the next question will say 'and were there any problems?'. If I had said 'yes' then it's asking me to tell me what the problems were. [...] so for every positive* (answer) *the next one* (question) *will come up. [...] See how you tick and then it tells you what bloods. So... Because you've ticked 'yes, he's high blood pressure', 'yes he's on treatment', the guideline's there: 'patients with hypertension': the minimum they're going to do is full blood count, U &Es, and ECGs. [...] If they have allergies, it opens up another form, you do all the recordings and everything. That's what I was saying: select the admission based on the nursing criteria. The bloods that was done... The dates it was done. And then we can write free text to say... and then the nurse has decided on a same-day admission."*

9. Protocol-based assessment:

- **POA nurse 1** :*"[...] This is much more thorough. It's more robust 'cause the guidelines are written into it, so it's not open for... – more robust in the way...– interpretations or [...] it's telling you there: 'if they're on high-blood pressure medicine this is what you do.' [...] when we used to do the paper copy with certain guidelines and protocols, then... So you did a day-surgery protocol, same-day admission protocol and inpatient protocol and if you answered 'yes' to everything, you looked at your protocol and it would tell you what to do. Now you don't have separate guidelines. The actually document is guidance in itself. Pre-op guidelines underpin the electronic e-form. So written within that... I'll show you when I'm doing it and you'll see. It's a really, really good system actually [...]*
But I would think the whole point – which probably is the biggest benefit

from electronic – is that we're all working to the same... it's standardized work. You're not looking and there's a different pre-op document in Gartnavel from what there is at the Royal. The patient's been assessed in the Royal but they're saying: 'that's not the documentation we use here' and the patient's getting surgery here. It's the same document. People were working different guidelines, so some people wouldn't do a patient if their body mass was above 35, but if you're assessing them at the Royal they did get it. [...] it's all about kind of standardizing the practice. Obviously you have to assess everybody individually – I am not meaning it like that – but the guidance is there for your base to use"

10. Screening tests and investigations:

- **POA nurse 1** : *"[...] you've got blood tests, ECG... they could get X-ray, could get an Echo* (-cardio-gram)*... We obviously do BP* (blood pressure)*, pulse, height, weight, BMI* (Body Mass Index)*or the usual, so you're doing baseline recordings [...]*
We have a template for every patient that we've seen... we all write the bloods that we've done on the patient, whether we've sent them anywhere, ECG... We'll follow-up everything and we'll have a bit to say whether they need an anaesthetic referral or whether the bloods are all signed, and referred and in the case-notes. So we'll follow-up all the tests that we do, we'll follow them up because that's part of the assessment, the information to say that we've really done that.[...] And we'll do MRSA screening if needed as well, so that's another thing we do[...] We know... What patients will get one 'cause there's a criteria and there's guidelines [...] the pre-op e-form has the guidelines underpinning it [...] and we can add them just by colour-coding them, so we can have lists, I've got any of them are MRSA screened, any that are waiting anaesthetic reviews. Any of that note to follow-up the blood results so that I can just go in and I don't need to be putting... it's just an easier way..."

11. Anaesthetic review:

• **POA nurse 1** : *"[...] the documentation identify if the patient requires an anaesthetic referral, so we would only refer direct to the anaesthetist who's doing the list. [...] we'd do a direct referral to the anaesthetist.*
Interviewer: " and that's through the electronic system as well?"
• **POA nurse 1** : *"Yep*
Interviewer: "...Direct referral. And then what happens? Do the patients see the anaesthetists?"
• **POA nurse 1** : *"Well no, they might not, it might just be advice that they give or they might want a further test done or they may say: 'no, that's fine to come in'. And we would go and then update the documentation... So that the patient knows... Sometimes patients have to be cancelled or sometimes they have to be deferred because maybe they want a cardiology review, or they want a patient to have an echo, and that would be a wee bit longer. It depends on the time scale we've got between the date that you're assessing the patient and whether they've got a TCI which is a 'To-Come-In' date. So we could be assessing somebody the day that he's going for surgery on the morning. So we don't have much time."*

12. Managing surgical admissions

• **POA nurse 1** : *"[...] Well sometimes the patient's already been scheduled as an inpatient but when we do the assessment we have to say what we feel the admission should be by assessment. And if we thought that this patient was suitable for day-surgery we tick that and the same as we feel they're suitable for same-day, we will tick that as well so its' based on the nursing assessment we've made."*
"[...] if we have to change their admission: for somebody's been booked for a day-surgery procedure but they're insulin-dependant diabetic and their diabetic control's not brilliant, we would probably phone the secretary and get that patient changed to an inpatient admission. So we can do different things like that... It's not all about just anaesthetic referrals, we need to take into consideration social aspects as well. Because if the patient lives alone, and they're really should go for day surgery because they need to have an adult with them overnight, so different things like that [...] the whole service is changed. At first, when I started, we only did day-cases,

we didn't assess inpatients. They got done when they went in to the wards the day before. They were done by the doctor and seen by the anaesthetist. It's just been a gentle progression [...]

- **Anaesthetist 1**: *"[...] I think the benefits which will... are starting to happen at... – which would happen if there's probably more investment resources throughout the hospitals – ...that patients are fully prepared now for surgery, their... All the correct information allows for better scheduling 'cause we now know, you know... Patients who are admitted generally won't be cancelled because of poor preparation. It also means that patients are now able to just come in on the day of surgery. You could argue that there will be few people admitted the day before surgery... So that sorts bed occupancy issues... and that's the main sort of efficiency [...] If someone's cancelled now [...] that will be reported and it will be looked into. Very few people are now cancelled on the day of surgery, because of poor preparation."*

13. Issues associated with accessing patients' case-notes:

- **POA nurse 2**: *"...a lot of the time the case-notes are missing for patients so if we didn't have patient case-notes, all the information is on the portal that we need. Whereas with the paper copy, if we didn't have the case-notes, you had no information, 'cause we didn't have that facility."*
- **POA nurse 3**: *"like in Golden Jubilee* (i.e. a special health-board), *they've got a separate set of case-notes. And so sometimes, if they've had echo tests which aren't yet on the Portal and the anesthetist just want to see it, you have to get the case-notes sent over separately. And that's not always fast. And again (name) had problems the other week 'cause there was a patient whose case-notes were in Aberdeen... had case-notes in Aberdeen. So she had to write a letter to say what she... what information she needed. She had to fax it through, wait for them to put it through all their processes and then fax through the information. But so: that's when it becomes a problem. Within the hospital and within even West Glasgow, or Greater Glasgow, it's not too big a problem. [...] but where it becomes a problem is when it's outwith Greater Glasgow"*

• **POA nurse 4**: *"Sometimes getting case-notes can be a problem... Sometimes you don't have the case-notes in time so you have to see patients without case-notes and obviously nowhere to file your assessments so you have to hold onto that information (*paper clinical notes*) until you get the case-notes to file it in [...] Well, it's not so much the information because you can get most of the information on the portal, but not having the case-notes... you can't file, you can't file their ECG or your assessment anywhere, you have to hold onto that paper-work until you can get access to the notes."*

14. Nurses' opinions on the main benefits of the electronic integrated care pathway:

(asked about the main perceived benefits of the POA ICP) • **POA nurse 1** : *"More than anything the fact that it's stored electronically so... If the pre-op document went missing or anything happened... The clinical portal is one of the best tools out. The clinical portal now has most information regarding the patient in it and from the clinical portal is when we... where we access the eForms. So it's all in the... done now when we go to the electronic patient records [...]*
It's much, much better because [...] you 've got all that information saved electronically, so you've always got that record there."

• **POA nurse 2**: *"It's great that it links up into the labs across the city and also... all kind of areas, it links up to... so we can access maybe from the GP surgery, we can access their referrals, we can access all the labs from... Even if the patients have had blood taken from their GP practice, we can get all of that. Also there's new things coming on, maybe on a... kinda monthly basis like... so that Echo reports: we're able to get that. We're also able to access any appointments that the patients are attending, so that gives us a bit of insight into these patients' history as well. So if they've maybe been attending respiratory* (department)*, you obviously think to yourself: this patient's got a respiratory problems or cardiac... so I think it's fantastic. And it's also great for us been able to email the anaesthetists and things like that as well."*

15. Nurses' opinions on the main dis-benefits of the electronic integrated care pathway:

- **POA nurse 2**: *"...With the paper copy, though, it was quicker. The electronic guidelines take a bit more time to open all the forms and things like that, a bit more time consuming... but it is better[...] It's just opening up individual forms, but that's just been taken away recently, actually, so it's a bit quicker and also if maybe there's IT problems: like computers are running slow, things like that... It takes a wee while to kind of go up and running... or if the printer's not working. These are all more time consuming things. More IT issues rather than with the paper work itself..."*
- **POA nurse 3**: *"I think, the electronic records takes slightly longer than the paper record because sometimes there are drop down boxes that don't really... apply"*
- **POA nurse 4**: *" The only disadvantage I can think of really is when the system goes down... Occasionally that happens and then we have to revert to a paper copy"*

16. Impacts on patient management:

- **Interviewer:** "[...] You do pre-op assessments for all specialties?"
- **POA nurse 1** :*"...Most specialties and patients that are going to all different hospitals, not even just in the north, so the whole of Greater Glasgow and Clyde use the same, and it's generic pre-op assessment, they use the standardized preop assessment guidelines which is electronic, so it's transferable across... whatever hospitals, so I could be working in the Royal and assessing a patients who is going to the Southern General and even though that's Southside, certainly the preop is valid for there... [...] so the patient may get seen in the Royal Infirmary by a Royal Infirmary surgeon [...] but then it happens to be that they use the theatres over in the Southern, so they're really a Glasgow Royal Infirmary patient or Gartnavel patient or Stobhill patient, so we do the pre-op assessment 'cause it's our patient and then it will go to... the Southern General [...] so that's how it's got to be transferable because it's where the surgeon has his operating list. [...] So they're assessed under the special... you know that specialty that the consultant with the hospital that they're in. So, certain surgeons*

work in Stobhill, they will be assessed in Stobhill even though their patients get their surgery done in Gartnavel. And the same for the Royal as well [...]

(asked about the main perceived efficiencies in the service)

• **POA nurse 3**: *"I don't think there's many patients coming on the day of surgery now and they have to be sent home without having their surgery done. So I think the utilisation point of view, I think that it's really improved that. [...]. Anaesthetists are well informed, now about patients coming in with... if they've got airway issues, the anaesthetist knows about... knows about it ahead of time so you can prepare for it, if they've... patients have chronic problems, then the anaesthetists again are well aware of them and can prepare as such or ask us to get other things done before they come in. So I think the time is utilised so much better because we're able to filter through the patients"*

• **POA nurse 4**: *"Another good thing is that when you go in (*the portal*) if somebody's seen the patient before – if it's may been another hospital – they'll already be an assessment in there. And you can look at that and take from that, that can speed up your assessment if somebody's seen them before. [...] Well you maybe just kind of confirm that everything is still as it was at the time of the last assessment. But it's still a lot quicker than doing the whole thing from the beginning [...] say may be (*nurse name*) had seen the patient 6 month previously, we would just update that document" "you've maybe seen a patient and there's been a referral and all this been done and you've sent the notes away and then maybe the following week, one of the secretaries or somebody phones asking you something about this patient. With the paper copies, they were away: you had nothing to look back on whereas with the portal you can go in and say yeah, that's right, this was done or no this wasn't [...] You can access it* (the eForm or record) *anytime after the event. You just need to go in and put in the patient's CHI number."*
